# Investigation of Genetic Factors and Clinical Data in Breast Cancer Highlights the Importance of Breastfeeding and Cancer History

**DOI:** 10.3390/cimb45100501

**Published:** 2023-09-28

**Authors:** Amanda Mercês, Rebecca da-Silva-Cruz, Caio S. Silva, Rommel Burbano, Ândrea Ribeiro-dos-Santos, Giovanna C. Cavalcante

**Affiliations:** 1 Laboratory of Human and Medical Genetics, Graduate Program in Genetics and Molecular Biology, Federal University of Pará (UFPA), Belém 66075-110, Pará, Brazil; amanda.merces@icb.ufpa.br (A.M.); rebeccalais94@gmail.com (R.d.-S.-C.); scaio@hotmail.com (C.S.S.); akelyufpa@gmail.com (Â.R.-d.-S.); 2 Laboratory of Molecular Biology, Ophir Loyola Hospital (HOL), Belém 66063-240, Pará, Brazil; rommel@ufpa.br

**Keywords:** breast cancer, apoptosis, Amazon, *TP53*, *CASP3*, *CASP9*, *BCL2*

## Abstract

Breast cancer (BC) is the type of neoplasm that most affects women worldwide. It is known that one of the hallmarks of cancer is the resistance to cell death with the evasion of apoptosis. Considering the relevance of *TP53*, *BCL2*, *CASP3,* and *CASP9* genes for the occurrence of the intrinsic apoptosis, this study investigated the distribution of the genetic variants rs17880560 (*TP53*), rs11269260 (*BCL2*), rs4647655 (*CASP3*), rs4645982, and rs61079693 (*CASP9*), as well as genetic ancestry and clinical data, in a BC cohort from the Brazilian Amazon that other variants in these genes might play a role in this process. In the present study, 22 breast cancer tissues and 10 non-cancerous tissues were used, therefore, 32 samples from different patients were subjected to genotyping. We observed that breastfeeding and cancer history were factors that need to be considered for BC (*p* = 0.022). Therefore, this study contributed to a greater understanding of intrinsic apoptosis in BC, reinforcing previous data that suggest that the history of cancer might be a condition that affects the development of BC and that breastfeeding may act as a protective factor for this type of cancer. We recommend more studies on the genetic factors investigated here, aiming at a future with tools that can help in the early diagnosis.

## 1. Introduction

Breast cancer (BC) is the tumor type that most affects women worldwide; according to the International Agency for Research on Cancer [1], BC obtained the incidence of 2.26 million cases worldwide by 2020, with a projection for 2.67 million new cases in 2030 for women. In addition, mortality might rise from 685,000 deaths by 2020 to 857,000 by 2030, an increase of 25%, according to the same estimates. In Brazil, it was estimated that between 2020 and 2022, 66,280 cases of BC in women per year were diagnosed, being an estimated risk of 61.61 new cases per 100,000 women [2].

Breastfeeding has already been described as a factor that decreases the risk of breast cancer [3,4]. Thus, apparently the earlier the breastfeeding or the greater the number of children breastfed, the greater this protective effect will be. It is suggested that breastfeeding for at least one year reduces the risk of developing breast cancer by 48%, and the twelve months of breastfeeding need not to be continuous [3]. Differentiation of breast tissue only occurs during the lactation period, therefore, breast cells from a woman who has never breastfed may be more vulnerable to carcinogens that induce cellular mutations and transformation into neoplastic cells [3]. Lactation also inhibits ovulation and the stimulation of ovarian hormones, decreasing the mitogenic effect of estrogen [4].

BC can be highly heterogeneous; it can be aggressive, in addition to being responsive or not to certain treatments [5,6]. BC encompasses several subtypes with distinct biological, molecular, and clinical results. Due to the clinical complexity and heterogeneity of breast cancer, it has become necessary to establish classification systems that standardize the type of tumors for prognostic and therapeutic benefits. Thus, currently, breast cancer is subdivided into different molecular types through immunohistochemical studies, in correspondence with gene expression assays (BC molecular classification).

In this way, knowledge about the molecular classification of BC is essential for the best therapeutic targeting of the disease [5,6]. BC is classified into four molecular subtypes (luminal A, luminal B, HER2+, and triple-negative/basal-like), for which the presence of some receptors in the tumor cell membrane in the analyzed biopsy through the immunohistochemistry is considered as a grouping criterion [7]. The luminal A molecular subtype is characterized by the presence of estrogen (ER) and progesterone (RP) receptors, and a low cell proliferation index measured by the ki67 marker, in addition to not having amplification of the ERBB2 gene (HER-2 protein). Among the molecular types, it is the subtype with the best prognosis among breast cancer patients. The luminal B subtype also presents hormone receptors, but with a proliferation index greater than or equal to 14% and is negative for HER-2, in addition to having a more guarded prognosis when compared to luminal A. The third molecular group includes cells which only have the HER-2 receptor and variable Ki67. This type of cancer represents a more guarded prognosis in relation to the luminal subtypes. Finally, the triple-negative (TN) subtype is characterized by the absence of ER, PR, and HER2 and differs from normal cells by having an elevated Ki67 index. They are biologically more aggressive and represent the worst prognosis among the groups mentioned [7]. Additionally, malignant tumors are classified according to the rules established by the Union for International Cancer Control (UICC) [8], from the classification of malignant tumors (TNM), which considers the size of the tumor (T), presence and extent of regional lymph node involvement (N), and the presence of distant metastasis (M). To prevail in the organism and progress to metastasis, the tumor must acquire some characteristics, such as cell death resistance [9].

Normal cells are responsive to cell signaling that leads to apoptosis; on the other hand, cancer cells can evade this system, enabling the permanence of the damaged cell [10]. The p53 protein is responsible for numerous cellular processes, including the regulation of the BCL-2 family proteins, which are related to cell membrane permeability and intrinsic apoptosis. Intrinsic apoptosis, also called the mitochondrial pathway, is regulated by members of BCL-2 family, leading to the activation of initiator caspases, including CASP9. The apoptotic pathways will converge in the executor phase, in which a class of proteins called executing caspases, such as CASP3, will mediate the proteolytic breakdown of the cell [11]. Therefore, the *TP53*, *BCL2*, *CASP3,* and *CASP9* genes are important for the occurrence of this type of cell death.

The purpose of the present study was to investigate variants in genes related to the intrinsic pathway of apoptosis, correlating with clinical data in breast cancer. For that, we analyzed the allele and genotype distribution of rs17880560 (*TP53*), rs11269260 (*BCL2*), rs4647655 (*CASP3*), rs4645982, and rs61079693 (*CASP9*) in BC patients and cancer-free individuals from northern Brazil in search of new biomarkers for this neoplasm.

## 2. Materials and Methods

### 2.1. Sampling

All samples used in the study were recruited according to ease of access. Surgical specimens were obtained by excising the lesion, and the materials were fixed in 10% buffered formalin shortly after collection. The samples used were fresh frozen tumor tissues (in cases of breast cancer) and fresh frozen tissues without cancer. Cancer-free tissue samples were from voluntary patients undergoing mammoplasty. As previously mentioned, diagnosed breast cancer samples were selected by convenience, thus, after the diagnosis of the disease by a pathologist, it was selected for the study with agreement of the patients. Thus, the inclusion criteria included cancer diagnosis. Likewise, for samples without cancer, confirmation of cancer-free tissue by a pathologist took place, including individuals with no diagnosis of cancer and excluding individuals with cancer history.

Thirty-two samples divided into two groups were investigated: 10 samples from individuals without cancer and 22 samples from individuals with breast adenocarcinoma. Samples were obtained from Hospital Ophir Loyola, Brazil. Clinical data (age, history of cancer, breastfeeding, number of births, age at menarche, presence or absence of menopause, smoking, alcoholism, and tumor classification) were provided by expert physicians and informed consent was obtained from all subjects involved in the study.

### 2.2. DNA Extraction and Quantification

DNA extraction was performed based on phenol/chloroform/isoamyl alcohol protocol [12]. DNA quantification was performed with a NanoDrop 1000 spectrophotometer (Thermo Fisher Scientific, Waltham, MA, USA). Estimation of the DNA concentration of the extracted samples was performed at an absorbance of 260 nm. DNA quantification was performed on all samples before the PCR step.

### 2.3. Genotyping

Five apoptosis markers present in a set previously described by our research group [13] were used. The markers are INDEL-type polymorphisms. Multiplex PCR was performed to amplify all markers in a single reaction, with 5 μL of QIAGEN Multiplex PCR Master Mix (QIAGEN, Hilden, Germany), 1.0 μL of Q-solution, 1.0 μL of water, 1.0 μL of primer mix, and 2.0 μL of DNA per sample. For the amplification reaction, a Veriti thermal cycler (Thermo Fisher Scientific) was employed, with the following protocol: 95 °C for 15 min, followed by 35 cycles of 94 °C for 45 s, 60 °C for 90 s, and 72 °C for 1 min, with final extension at 70 °C for 30 min. Additionally, genomic ancestry analysis was performed with a panel of 61 markers previously described by our research group [14,15]. For this, multiplex PCR was performed with the following protocol: 5.0 μL of QIAGEN Multiplex PCR Master Mix, 1.0 μL of Q-solution, 1.0 μL of Primer Mix, 2.0 μL of water, and 1.0 µL of DNA. PCR was performed with the following cycling program: 95 °C for 15 min; 35 cycles 94 °C for 45 s, 60 °C for 90 s, and 72 °C for 60 s; 70 °C for 30 min and for the subsequent analysis of fragments, the protocol of 7 μL of ultra-pure formamide + Liz was used, with the proportion of 8.7 of formamide and 0.3 of LIZ and 3 μL of multiplex PCR product, at the sequencer ABI 3130 (Thermo Fisher Scientific).

### 2.4. Statistical Analysis

Statistical analysis was performed using the JASP v. 0.9.2.0, considering statistically significant values with a *p*-value ≤ 0.05. We used an ANOVA to analyze different groups, in addition to a Chi-squared test corrected by the Bonferroni method to compare our clinical data. We used a logistic regression to estimate odds ratios (OR) at 95% confidence intervals (CI) and *p*-value in the analysis of the investigated polymorphisms. Genomic ancestry was analyzed using an internal tool of the research group based on Structure v. 2.3.4 and the *p*-values were obtained by Mann–Whitney test.

## 3. Results

### 3.1. Characterization of the Sample Population

The cohort was composed of 32 Brazilian women: 22 in the case group and 10 in the control group. Clinical aspects such as age, history of cancer, breastfeeding, number of births, age at menarche, presence or absence of menopause, smoking, alcoholism, and tumor staging based on TNM classification were considered for the case group (Table 1).

**Figure 1:**
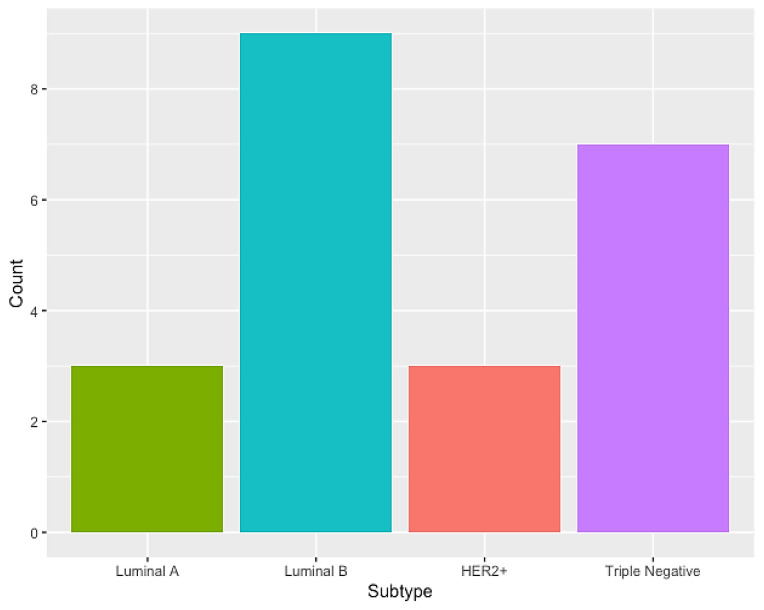
Distribution of molecular subtypes of breast cancer.

Additionally, the analysis of clinical data regarding the TNM staging was performed, as shown in Table 2. Interestingly, history of cancer and breastfeeding had the same frequency of 22.7% (*p*-value = 0.022). As for the molecular subtypes, luminal B was notably the most frequent in our cohort (Figure 1).

**Figure 2:**
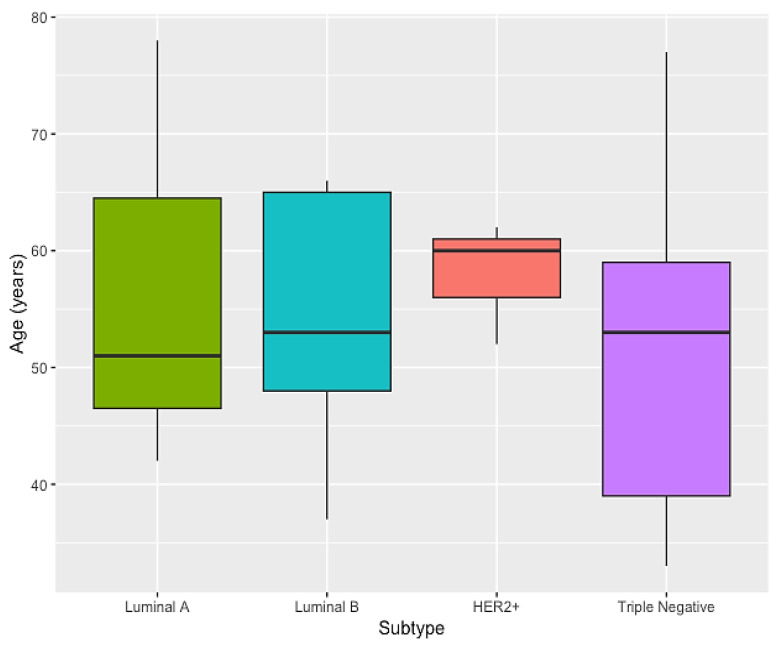
Age distribution within the different breast cancer subtypes.

We also analyzed age in relation to the molecular subtypes of BC and observed that the HER2+ subtype had a higher mean age when compared to the other molecular subtypes (*p* = 0.862) (Figure 2). The HER2+ subtype had an average age of 58 years, while the luminal A, luminal B, and triple-negative subtypes had an average age of 57, 54.4, and 51.2 years, respectively.

**Figure 3:**
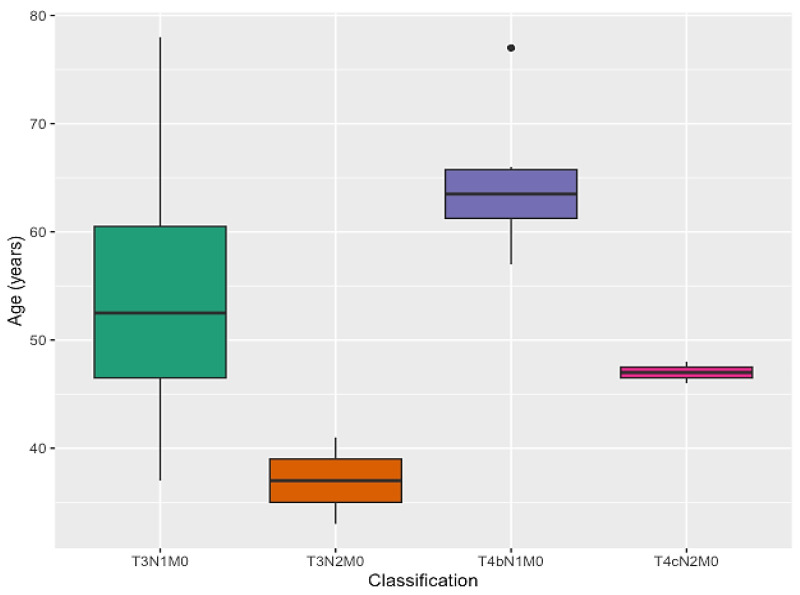
Age distribution by TNM staging of breast cancer in the studied cohort.

When we evaluated tumor staging and other variables (Table 2), approximately 54% were in T3N1M0, and the mean age was 53.1 years (Figure 3); 27% were in T4N1M0, with a mean age of 64.6 years; 9% were in T3N2M0, with a mean age of 37 years; and 9% in T4N2M0 with a mean age of 47 years (*p* = 0.01).

### 3.2. Selection of Investigated Polymorphisms

Five INDEL-type polymorphisms from a genetic panel previously reported by our research group [13] were investigated. Such markers are described in Table 3. For more information on the genetic panel, please refer to our previous work [13].

We compared the distribution of deletion/deletion (DEL/DEL), insertion/insertion (INS/INS), and insertion/deletion (INS/DEL) genotypes for each polymorphism between both groups. These data are described in Table 4. We did not find statistically significant differences in our cohort, which suggests that such polymorphisms may not be associated with the development of breast cancer.

### 3.3. Analysis of Genomic Ancestry

Regarding genomic ancestry, when analyzing only case and control, we found no statistical significance, as seen in Table 5. Our data demonstrate that both case and control groups have a close European ancestry contribution, with 0.64 and 0.67, respectively. Regarding the African contribution, we observed 0.21 for the case group and 0.08 for the control group (*p* = 0.122). As for Native American ancestry, an average of 0.14 and 0.26 was observed in the case and control groups (*p* = 0.247). However, there were no statistically significant differences between the contributions, possibly due to the small sample size.

We also performed genetic ancestry analysis according to BC molecular subtypes (Table 6). Regarding European ancestry, we obtained a result of 0.35, 0.76, 0.52, and 0.66 being the subtypes luminal A, luminal B, HER2+, and triple-negative (TN), respectively (*p* = 0.3845). For African ancestry, the results were 0.45, 0.11, 0.26, and 0.20 (*p* = 0.2408); regarding Native American ancestry, the observed values were 0.20, 0.12, 0.20, and 0.13, respectively (*p* = 0.759).

As for the tumor subtypes, we observed that luminal B, HER2+, and triple-negative mostly had European ancestry and that luminal A had the highest African ancestry contribution. When we analyzed African ancestry, we noticed that luminal A and B had significantly different ancestry contributions, since luminal A had 0.450 and luminal B had 0.117. The triple-negative subtype had an average of 0.66 when analyzing European ancestry and 0.20 regarding African ancestry.

## 4. Discussion

In this study, we focused on investigating INDEL variants in genes related to apoptosis in BC. We identified the genotypic distribution in the *BCL2*, *CASP3*, *CASP9,* and *TP53* genes, comparing case and control groups, staging, and molecular subtype. In addition, we compared several clinical aspects that may influence the appearance of the tumor, such as smoking, alcoholism, cancer history, and reproductive factors like age at menarche, menopause, breastfeeding, and others.

Age is an important factor for the occurrence of the disease, since the largest number of women affected by BC grows from the age of 40 [16]. These results were also found in a previous study [17], in which 55.4% of women who developed breast cancer were diagnosed before age 60, with a mean age at diagnosis of 53 years. Our findings corroborate these data, since the mean age of the case group was approximately 54 years.

When analyzing breastfeeding in relation to tumor staging, we observed that 77% of the women in the control group did not breastfeed (*p* = 0.022). Breastfeeding is an important variable to be considered, as it has been observed that women who breastfeed had a reduced risk of BC [18]. A study carried out with elderly women [19] found that the longer the duration of breastfeeding, the greater the protection against cancer. Differentiation of breast tissue only occurs during lactation. In our clinical data, most women in the case group did not experience breastfeeding, which suggests that this factor may have contributed to greater exposure of breast tissue to carcinogens and to the occurrence of mutations that predisposed to a malignant tumor [20].

Regarding the history of cancer, 77% reported not having any history of the neoplasm (Table 1). When analyzing the cancer history in relation to the stage of the disease (Table 2), we found a statistically significant correlation (*p* = 0.022). Therefore, there is a possible relationship between cancer history and tumor staging based on our findings, corroborating a study carried out in the United Kingdom with approximately 113,000 women, which found that those who had a first-degree relative with BC had a 1.75 higher risk of developing this type of cancer [17].

Regarding the molecular distribution of BC, our profile is similar to another study carried out in Brazil [7], which showed a 59% prevalence of the luminal B subtype, which is also the majority in our findings (41%). However, here, the triple-negative subtype was the second most frequent (32%); these data differ from most studies [7,21], in which the triple-negative subtype is commonly the least frequent. In this sense, identifying the subtype of BC is fundamental for better management of the disease and inferring the behavior of the tumor.

As for the investigated polymorphisms, we found no statistical significance in our sample, suggesting that other variants in these genes might influence BC. The literature in respect to these variants in *BCL2*, *CASP3*, *CASP9*, and *TP53* genes is scarce, especially regarding BC; one study from our research group sought to associate these variants with gastric cancer in the Brazilian population, with no statistical significance [13]. Hence, this is the first study to investigate such polymorphisms in BC.

The *BCL2* gene is an intrinsic regulator of apoptosis and over 60% of BC patients have elevated levels of the BCL2 protein [22]. In a study conducted in India, a variant of the *BCL2* gene (rs2279115) was found to contribute to the risk of developing non-small cell lung cancer [23]. We noticed, therefore, that mutations in this gene may be related to other types of cancer, but not necessarily to the polymorphism investigated in the present study.

*CASP3* is a gene that encodes the homonymous protein and is responsible for executing apoptosis (executor phase). There is evidence that mutations in caspases play a unique role in the pathogenesis of malignant tumors and most caspases detected in these tumors demonstrate reduced apoptotic activity when compared to wild-type caspase [24].

In previous studies with *CASP9* and *CASP10* polymorphisms, including rs4645982 (*CASP9*) [25], the association with cancer susceptibility was sought, which did not find a significant association of this variant with the incidence of cancer in different inheritance patterns. However, other *CASP9* polymorphisms (rs4645981 and rs1052571) have been associated with the risk of developing cancer. Furthermore, a study with the *CASP9* polymorphism (rs4645982) and acute myeloid leukemia (AML) [26], also did not find a significant *p*-value for the susceptibility (*p* = 0.193). In this sense, our results corroborate these studies for rs4645982 in different types of cancer.

As for the rs17880560 (*TP53*) polymorphism, there was no association of this polymorphism on BC in the investigated sample from northern Brazil. However, research carried out in the Brazilian states of São Paulo and Rio Grande do Sul [27] found that this variant had a relative risk of 3.17 (95% CI: 1.83–5.17) of development of cancer before the age 25. The *TP53* gene is widely studied; for instance, a recent work that sought to verify mutations in this gene found that more than 91% of tumors with mutations had structural loss of both alleles, which may contribute to genomic instability and progression of cancer [28]. Furthermore, a review produced from database analysis found that breast and ovarian cancer share a high frequency of mutations in *TP53*, most of which are frameshift mutations [28], indicating that such mutations can serve as potential biomarkers for these types of cancer.

In short, among the polymorphisms investigated in this study, only rs17880560 (*TP53*) was associated with another study with a positive result for the appearance of cancer at an early age. Regarding genomic ancestry, although we did not find statistically significant results in our study (*p*-value = 0.1668), knowledge of the ancestry of patients diagnosed with BC is important to verify possible factors that corroborate a worse prognosis, in addition to direct treatment with a focus on improving clinical outcomes for patients.

## 5. Conclusions

This study contributed to a better understanding of intrinsic apoptosis regarding breast cancer. Based on our results, we suggest that other polymorphisms of the studied genes (*BCL2*, *CASP3*, *CASP9*, and *TP53*) may be associated with breast cancer, or that studies polymorphisms should be carried out in larger cohorts. In addition, we reinforce the results already seen in the literature about the contribution of breastfeeding as a protective factor for BC. Although more studies are needed to strengthen our findings, due to our limited sample size, this work contributed to the knowledge of genes and INDEL variants of apoptosis and their relationship with breast cancer.

## Figures and Tables

**Table 1:** Clinical data of patients with breast cancer based on molecular classification.

Clinical Data		%	*p*-Value
Age	30–55	54.4	
	>55	45.4	
	Mean	54.3	0.862 ^1^
Cancer history	Yes	22.7	0.337
	No	77.3	
Breastfeeding	Yes	22.7	0.337
	No	77.3	
Number of births	1–2	54.5	0.901
	3–5	45.4	
Age at menarche	10–13	59	0.151
	14–17	41	
Menopause	Yes	50	0.193
	No	50	
Smoking	Yes	18	0.729
	No	82	
Alcohol Consumption	Yes	9	0.365
	No	91	

^1^—*p* value obtained by ANOVA; other *p* values obtained by chi-squared test.

**Table 2:** Variables and distribution of a breast cancer cohort.

Variables		Sample Number	%	*p-*Value ^1^
Cancer history	Yes	5	22.7	0.022
	No	17	77.3	
Breastfeeding	Yes	5	22.7	0.022
	No	17	77.3	
Number of births	1–2	12	54.5	0.128
	3–5	10	45.4	
Age at menarche	10–13	13	59	0.375
	14–17	9	41	
Menopause	Yes	11	50	0.261
Smoking	Yes	4	18	0.736
Alcohol Consumption	Yes	2	9	0.843
T3N1M0		12	54.5	0.288 ^2^
T3N2M0		2	9	
T4N1M0		6	27.5	
T4N2M0		2	9	

^1^—*p* value obtained by chi-squared test. ^2^—*p* value obtained by ANOVA.

**Table 3:** Characterization of the investigated apoptosis markers.

Gene	ID	Region	Alleles
*BCL2*	rs11269260	INTRON	TCTATCACCGATCATT/−
*CASP3*	rs4647655	INTRON	−/AAATCCTGAA
*CASP9*	rs4645982	INTRON	−/TCCCCGCACTGACCTCCACG
*CASP9*	rs61079693	INTRON	AAAA/−
*TP53*	rs17880560	3′ UTR	−/GCCGTG

**Table 4:** Genotype and allele distribution of the investigated variants in case and control groups. OR—odds ratio, CI*—*confidence interval. *p*-value, OR, and CI were obtained with logistic regression.

Genes	Genotype	Case (68.75%)	Control (31.25%)	*p*-Value	OR (CI 95%)
***BCL2***	**rs11269260**				
	DEL/DEL	6 (30%)	4 (40%)	0.455	0.444 (0.053–3.738)
	INS/DEL	12 (60%)	4 (40%)	0.305	2.250 (0.478–10.595)
	INS/INS	2 (10%)	2 (20%)	0.585	0.643 (0.132–3.140)
	Deletion allele	0.6 (60%)	0.6 (60%)		
	Insertion allele	0.4 (40%)	0.4 (40%)		
***CASP3***	**rs4647655**				
	DEL/DEL	10 (53%)	4 (40%)	0.433	1.875 (0.390–9.013)
	INS/DEL	7 (37%)	5 (50%)	0.354	0.467 (0.093–2.339)
	INS/INS	2 (10%)	1 (10%)	0.965	1.059 (0.084–13.329)
	Deletion allele	0.72 (72%)	0.50 (50%)		
	Insertion allele	0.28 (28%)	0.50 (50%)		
***CASP9***	**rs4645982**				
	DEL/DEL	3 (23%)	3 (37.5%)	0.481	0.500 (0.073–3.435)
	INS/DEL	6 (46.2%)	3 (37.5%)	0.698	1.429 (0.236–8.637)
	INS/INS	4 (30.8%)	2 (25%)	0.777	1.333 (0.183–9.725)
	Deletion allele	0.46 (46%)	0.625 (62.5%)		
	Insertion allele	0.54 (54%)	0.375 (37.5%)		
	**rs61079693**				
	DEL/DEL	4 (25%)	1 (16.7%)	0.926	0.909 (0.123–6.715)
	INS/DEL	10 (62.5%)	3 (50%)	0.481	2 (0.291–13.738)
	INS/INS	2 (12.5%)	2 (33.3%)	0.306	0.308 (0.032–2.942)
	Deletion allele	0.625 (62.5%)	0.5 (50%)		
	Insertion allele	0.375 (37.5%)	0.5 (50%)		
***TP53***	**rs17880560**				
	DEL/DEL	9 (90%)	8 (47.1%)	0.172	0.281 (0.046–1.734)
	INS/DEL	1 (10%)	8 (47.1%)	0.073	8.000 (0.822–77.814)
	INS/INS	0	1 (5.8%)	0.994	3.383 × 10^−8^ (0.000–∞)
	Deletion allele	0.9 (90%)	0.53 (53%)		
	Insertion allele	0.1 (10%)	0.47 (47%)		

**Table 5:** Description of genetic ancestry contribution by case and control.

Genomic Ancestry	Case (N = 22)	Control (N = 10)	*p*-Value
European	0.643 ± 0.272	0.657 ± 0.313	0.730
African	0.211 ± 0.235	0.081 ± 0.132	0.122
Native American	0.147 ± 0.115	0.262 ± 0.221	0.247

**Table 6:** Genomic ancestry according to molecular subtypes of breast cancer.

Genomic Ancestry	Luminal A (N = 3)	Luminal B (N = 9)	HER2+ (N = 3)	Triple Negative (N = 7)	*p*-Value	*p-*Value (EU + AF + NA)
European	0.350 ± 0.473	0.762 ± 0.193	0.525 ± 0.120	0.665 ± 0.243	0.3845	0.1668
African	0.450 ± 0.382	0.117 ± 0.150	0.267 ± 0.226	0.205 ± 0.230	0.2408	
Native American	0.200 ± 0.095	0.122 ± 0.088	0.209 ± 0.215	0.130 ± 0.114	0.7590	

## Data Availability

The datasets used and/or analyzed during the current study are available from the corresponding author on reasonable request.
